# A first survey of the rye (*Secale cereale*) genome composition through BAC end sequencing of the short arm of chromosome 1R

**DOI:** 10.1186/1471-2229-8-95

**Published:** 2008-09-19

**Authors:** Jan Bartoš, Etienne Paux, Robert Kofler, Miroslava Havránková, David Kopecký, Pavla Suchánková, Jan Šafář, Hana Šimková, Christopher D Town, Tamas Lelley, Catherine Feuillet, Jaroslav Doležel

**Affiliations:** 1 Laboratory of Molecular Cytogenetics and Cytometry, Institute of Experimental Botany, Sokolovská 6, CZ-77200 Olomouc, Czech Republic; 2 INRA- Université Blaise Pascal, UMR GDEC 1095, 234 Avenue du Brezet, F-63100 Clermont-Ferrand, France; 3 University of Natural Resources and Applied Life Sciences, Department for Agrobiotechnology, Institute for Plant Production Biotechnology, Konrad Lorenz Str. 20, A-3430 Tulln, Austria; 4 The J. Craig Venter Institute, 9704 Medical Center Drive, Rockville MD 20850, USA; 5 Department of Cell Biology and Genetics, Palacký University, Šlechtitelù 11, CZ-78371 Olomouc, Czech Republic

## Abstract

**Background:**

Rye (*Secale cereale *L.) belongs to tribe Triticeae and is an important temperate cereal. It is one of the parents of man-made species Triticale and has been used as a source of agronomically important genes for wheat improvement. The short arm of rye chromosome 1 (1RS), in particular is rich in useful genes, and as it may increase yield, protein content and resistance to biotic and abiotic stress, it has been introgressed into wheat as the 1BL.1RS translocation. A better knowledge of the rye genome could facilitate rye improvement and increase the efficiency of utilizing rye genes in wheat breeding.

**Results:**

Here, we report on BAC end sequencing of 1,536 clones from two 1RS-specific BAC libraries. We obtained 2,778 (90.4%) useful sequences with a cumulative length of 2,032,538 bp and an average read length of 732 bp. These sequences represent 0.5% of 1RS arm. The GC content of the sequenced fraction of 1RS is 45.9%, and at least 84% of the 1RS arm consists of repetitive DNA. We identified transposable element junctions in BESs and developed insertion site based polymorphism markers (ISBP). Out of the 64 primer pairs tested, 17 (26.6%) were specific for 1RS. We also identified BESs carrying microsatellites suitable for development of 1RS-specific SSR markers.

**Conclusion:**

This work demonstrates the utility of chromosome arm-specific BAC libraries for targeted analysis of large Triticeae genomes and provides new sequence data from the rye genome and molecular markers for the short arm of rye chromosome 1.

## Background

Rye (*Secale cereale *L.) is a temperate cereal belonging to the tribe Triticeae, which is grown mainly in Europe and Northern America. Its uses include grain, hay, pasture, cover crop, green fodder, and green manure. More than 50% of the annual rye harvest is used for bread making, resulting in rich, dark bread that holds its freshness for about a week. Despite its relatively low acreage compared to other cereals, rye is of great importance due to its broad tolerance to biotic and abiotic stress, a feature generally lacking in other temperate cereals. Thus, rye remains an important grain crop species for cool temperate zones.

Besides its importance as a crop, rye is one of the parents of a man-made species Triticale and the short arm of rye chromosome 1 (1RS) has been introgressed into several hundreds of wheat cultivars [1,2]. In fact, some of the most successful wheat varieties carry the 1BL.1RS translocation as the presence of 1RS in the wheat genome increases both yield and protein content in grains [3]. Moreover, 1RS carries a cluster of genes encoding resistance to stem, leaf and yellow rust – Sr31, Lr26 and Yr9, respectively [4] and a self-incompatibility locus [5]. On the down side, 1RS carries the *Sec-1 *locus coding for ε-secalin, which negatively affects bread making quality [6]. Thus, it would be of great advantage to isolate those genes individually through map-based cloning and develop markers for marker assisted selection in rye and wheat.

Despite the economic importance of rye, little is known about its genetic make up at the DNA sequence level. To our knowledge, there is no ongoing sequencing project in rye, and there are no plans to target gene-rich fractions of its genome. Rye is underrepresented in the sequence databases compared to wheat and barley for which 1,104,002 and 529,839 sequences respectively, are deposited in GenBank. There are only 9,807 rye sequences (about 5 Mbp) available, of which about 90% are expressed sequence tags (ESTs). Updated list of rye genes, markers and linkage data was created by Schlegel and Korzun [7]. The lack of sequence information is a major limitation for marker development and gene cloning in this species.

The monoploid genome size of rye (1Cx = 7,917 Mbp) is the largest among temperate cereals, almost 40% larger than that of bread wheat (Table 1). This is due to the presence of a large amount of highly repetitive sequences. Flavell et al. [8] estimated the repetitive DNA content of rye to be 92%. Despite the progress in sequencing technology and bioinformatics, sequencing the whole rye genome remains a very difficult and expensive task. In particular, genome shotgun sequencing of such a large and repetitive genome seems currently impossible. On the other hand, the short arm of rye chromosome 1 represents only 5.6% of the rye genome and with the molecular size of 441 Mbp, 1RS is comparable in size to the whole rice genome, which was recently sequenced [9-11]. Recently, a method has been developed to dissect large plant genomes into individual chromosomes using flow cytometric sorting (reviewed in [12,13]). A protocol for sorting individual rye chromosomes has been set up by Kubaláková et al. [14], and Šimková et al. [15] created two BAC libraries from flow sorted 1RS arms. The library represents a valuable tool for map-based cloning, targeted sequencing and marker development.

**Table 1:** Genome size of major Triticeae species

**Species**	**Chromosome number**	**1C genome size**	1Cx genome size*	**Reference**
Rye	2n = 2x = 14 (RR)	7,917 Mbp	7,917 Mbp	[51]
Durum wheat	2n = 4x = 28 (AABB)	12,030 Mbp	6,015 Mbp	[52]
Bread wheat	2n = 6x = 42 (AABBDD)	16,937 Mbp	5,646 Mbp	[52]
Barley	2n = 2x = 14 (HH)	5,100 Mbp	5,100 Mbp	[51]

*Monoploid genome size (see [53] for terminology on genome size)

End sequencing of BAC clones enables generating random sequence information distributed across the whole genome. Kelley et al. [16] developed a protocol for high throughput BAC end sequence (BES) generation using automated sequencers. This protocol is now a routine in large sequencing centers, reducing cost and enabling the creation of large data sets. Nevertheless, the number of BESs from the Triticeae tribe is currently limited with only 37,609 hexaploid wheat BESs and 32 *Triticum monococcum *BESs in GenBank, representing the whole tribe. Beyond the sequence information itself, BESs are a valuable source of molecular markers. Shultz et al. [17] used BESs derived from BACs, representing minimum tiling path of soybean, to develop new microsatellite markers. Among the first 135 primer pairs tested, more than 60% were polymorphic. Paux et al. [18] took advantage of a BAC library specific for wheat chromosome 3B [19], and sequenced BAC ends to isolate chromosome-specific molecular markers based on inserted transposable elements (ISBP – Insertion Site Based Polymorphism). Paux et al. [18] succeeded in developing thirty-nine 3B-specific markers to anchor BAC contigs to the genetic/deletion map and have since then developed several hundreds of ISBP markers from 3B (unpublished). According to the authors' estimate, about 5% of BESs are suitable for ISBP development.

Here, we report on DNA sequence composition of the short arm of rye chromosome 1 (1RS) and on the development of new molecular markers for this chromosome arm using 2 Mb of BAC end sequences. We demonstrate that the combination of chromosome arm-specific BAC library with BAC end sequencing technology offers a cost efficient strategy to survey the composition of the rye genome and saturate chromosome 1RS with molecular markers.

## Methods

### Plant material

Seeds of rye (*Secale cereale *L., 2n = 14) cv. Imperial and wheat (*Triticum aestivum *L., 2n = 42) cv. Chinese Spring were kindly provided by Prof. A. J. Lukaszewski (University of California, Riverside, USA). Seeds of barley (*Hordeum vulgare *L., 2n = 14) cv. Akcent were obtained from Dr. P. Martínek (Agriculture Research Institute, Kroměříž, Czech Republic). A ditelocentric 1RS wheat-rye (Chinese Spring – Imperial) addition line (2n = 42 + 1RS'') was obtained from Dr. B. Friebe (Kansas State University, Manhattan, USA).

### 1RS-specific BAC libraries

Two BAC libraries specific for the short arm of rye chromosome 1 were constructed from DNA of 1RS arms, which were flow-sorted from the above mentioned wheat-rye ditelocentric 1RS addition line [15]. The SccImp1RShA library (*Hin*dIII) consists of 66,816 clones with an average insert size of 72 kb ordered in 174 × 384-well plates. The SccImp1RSbA library (*Bam*HI) consists of 36,864 clones with an average insert size of 75 kb ordered in 96 × 384-well plates. Collectively both libraries cover the chromosome arm 14-fold. As the 1RS arms were sorted from wheat alien chromosome addition line, the libraries contain about 14% of clones from various wheat chromosomes [15].

### BAC end sequencing

Two plates from each BAC library (plates SccImp1RShA_0079, SccImp1RShA_0127, SccImp1RSbA_0175 and SccImp1RSbA_0223) were chosen randomly for BAC end sequencing. DNA templates were prepared in 384-well format by a standard alkaline lysis method. The end sequencing was performed using Applied Biosystems (ABI) Big Dye terminator chemistry and analyzed on ABI 3730xl sequencer. Base calling was performed using TraceTuner and sequences were trimmed for vector and low quality sequences using Lucy [20].

### Annotation of sequences

Three repeat databases were used to analyze the repetitive fraction of the BAC-end sequences: TREPtotal [21,22], RepBase [23,24] and TIGR Plant Repeat Databases [25,26]. For identification of genes in BESs, 960,574 PUTs (PlantGDB-assembled Unique Transcripts) from various plant species were used. PUTs of *Arabidopsis thaliana *(143,848), *Avena sativa *(5,595), *Brachypodium distachyon *(9,924), *Glycine max *(102,265), *Hordeum vulgare *(103,345), *Oryza sativa *(153,740), *Secale cereale *(5,976), *Sorghum bicolor *(44,953), *Triticum aestivum *(243,326), *Triticum monococcum *(6,986) and *Zea mays *(140,616) were downloaded from PlantGDB [27,28].

### Identification of repetitive DNA elements

A semi-automated pipeline [18] was used to search for repetitive DNA elements in BAC-end sequences. The procedure involved two steps to find known repeats in a sequence and an additional step identifying potential repeats. In the first step, RepeatMasker [29] with the CrossMatch algorithm and default settings was used to search repeats without specifying a custom library. Thereafter, sequences were searched against TREPtotal, RepBase and TIGR Plant Repeat Databases. In the second step sequences were searched using TBLASTX (E-value = 1e^-5^) [30] against the same databases. Sequences matching known repeats were masked with an "X". Putative unknown repeats were identified by searching masked BESs with BLASTN [30] against themselves and 32,496 genome survey sequences (GSSs) of *Triticum *and *Aegilops *spp. downloaded from GenBank [31]. Sequences displaying 80% identity over at least 100 bp and five matches were assumed as unknown repeats and masked with an "X". The fraction of genome, represented by each repetitive DNA element, was calculated as ratio of cumulative length of sequences with homology to the element and the total length of BES data set.

### Gene content analysis

The repeat masked sequences were subjected to a homology search using BLASTN (E-value = 1e^-30^) against the PUT collections mentioned above. Cumulative match length was used to calculate the fraction of coding sequences in the rye genome as described for repetitive elements. Sequences matching PUTs coding for TE-related proteins were omitted. Sequences with alignment longer than 200 bp were searched using BLASTX against non-redundant protein sequences (with default setting except E-value = 1e^-10^).

### Development of molecular markers

We used SciRoKo 3.3 computer program [32] with default settings (except minimum score 14) for the identification of microsatellites in the BAC end sequences. BESs containing junction between two different sequences (repetitive elements or repetitive element and non-repetitive sequence) were identified from repeat-masking analysis for development of ISBP markers. Primer pairs were designed using PRIMER3 software [33] with default settings to border corresponding junction.

### Chromosome sorting and DNA amplification

**Figure 1:**
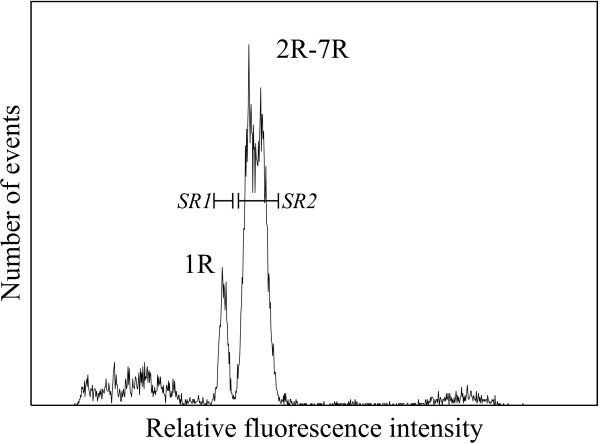
**Flow cytometric chromosome analysis and sorting in rye**. Histogram of relative fluorescence intensity obtained after analysis of suspension of DAPI-stained rye chromosomes. Peak of chromosome 1R is clearly resolved from the composite peak of chromosomes 2R-7R. Sorting regions were set to separate chromosome 1R (*SR1*) from other chromosomes (*SR2*).

Ten thousand 1R chromosomes were sorted from rye cv. Imperial according to Kubaláková et al. [14] into 20 μl ddH_2_O using a FACSVantage SE cell sorter (Becton Dickinson). Twenty thousand all rye chromosomes except 1R (2R – 7R) were sorted in the same way (Figure 1). Chromosomal DNA obtained after proteinase treatment was amplified using GenomiPhi DNA Amplification Kit (GE Healthcare, UK) according to the manufacturer's instruction to obtain 5 μg chromosome-specific DNA.

### Physical mapping of molecular markers

Sixty four ISBP primer sets were tested for 1RS specificity. For this purpose PCR was carried out on several DNA templates: a – rye (cv. Imperial); b – wheat (cv. Chinese Spring); c -wheat-rye (Chinese Spring – Imperial) telocentric 1RS addition; d – flow-sorted chromosome 1R; e – flow-sorted chromosomes 2R – 7R. Genomic DNA was isolated using Invisorb Spin Plant Mini Kit (Invitek) according to the manufacturer's instruction. The 10 μl standard PCR reaction contained 25 ng DNA, 1× PCR buffer, 0.01% Cresol Red, 1.5% sucrose, 0.2 mM each of dNTPs, 5 μM primers, 0.5 U Taq DNA polymerase. PCR was performed in a PTC-200 thermal cycler (MJ Research) as follows: initial denaturation at 95°C for 5 min; 35 cycles of 95°C for 30 sec, 62°C for 30 sec, 72°C for 30 sec; final extension for 5 minutes at 72°C. PCR products were separated on 2% agarose gel.

### Localization of a new repeat by Fluorescence in situ Hybridization (FISH)

To localize a newly identified repeat on mitotic metaphase chromosomes of rye, wheat and barley, root tips were collected to ice water for 26–30 h, fixed in a mixture of absolute alcohol: glacial acetic acid (3:1) at 37°C for seven days and stored at -18°C. Cytological preparations and *in situ *hybridization with labeled DNA were made according to Massoudi-Nejad et al. [34]. Digoxigenin-labeled probe was prepared from the newly identified COP1 repeat by PCR with specific primers (Left – CAACATTCGTGATGGTTTCG; Right – ATACACAAACCTGCCCCAAA). For identification of wheat homoeologous groups, reprobing with two additional probes was done. Biotin-labeled probe for GAA microsatellites was prepared using PCR with (GAA)_7 _and (CCT)_7 _primers and wheat genomic DNA as a template. A probe for 260-bp fragment of the *Afa *family repeat was prepared and labeled by digoxigenin using PCR with primers AS-A and AS-B on wheat genomic DNA according to Kubaláková et al. [35]. Chromosomes were counterstained with 1.5 μg/ml 4',6-diamidino-2-phenylindole (DAPI). Hybridization signal was visualized with anti-digoxigenin-fluorescein and Cy3-labeled streptavidin and observed under a fluorescence microscope (Olympus AX70 with a SensiCam B/W CCD camera attached).

## Results

### BAC end sequencing and sequence trimming

Four 384-well plates originating from two BAC libraries specific for rye chromosome arm 1RS were chosen to provide a uniform and random sample of 1RS. BAC clones were sequenced from both ends and after trimming, 2,778 (90.4%) useful sequences (longer than 60 bp) were obtained. In total, 2,032,538 bp of 1RS specific sequences were generated with average read length of 732 bp. These sequences represent 0.5% DNA of the short arm of rye chromosome 1. Analysis of the GC content of the sequenced fraction of 1RS is showed a 45.9% composition. All sequences were deposited in GenBank (Accession numbers FI104352–FI107129).

### Identification and characterization of repetitive DNA elements

The cumulative length of the sequences with homology to a certain repetitive element was used to estimate its representation in the rye genome. For example, sequences with homology to Copia retroelements had a cumulative length of 281,937 bp representing 13.9% of the BESs (2,032,538 bp). Should BESs composition be representative for rye, Copia elements account for 13.9% of the whole rye genome (Table 2).

**Table 2:** Representation of repetitive element groups on 1RS

			Cumulative length (bp)	Fraction of 1RS (%)
Class I elements			1,306,781	64.3
	LTR retrotransposons		1,275,443	62.8
		Gypsy	989,195	48.7
		Copia	281,937	13.9
		TRIM	265	0.0
	Non-LTR retrotransposons		31,338	1.5
		LINE	23,400	1.2
		SINE	1,877	0.1

Class II elements			100,854	5.0
		CACTA	90,323	4.4
		Mutator	1,394	0.1
		MITE	4,681	0.2
		LITE	2,753	0.1

Unclassified elements			30,340	1.5

Other known repeats			106,376	5.2
	Ribosomal genes		94,665	4.7
	Simple repeats		5,049	0.2
	Tandem repeats		6,662	0.3
Known repeats			1,536,587	75.6
Unknown repeats			178,027	8.8

Total			1,712,364	84.2

In total, 75.6% of the data set showed homology to repeats deposited in the databases listed above. Retrotransposons (Class I elements) were the dominant repeat group in the analyzed sequences, comprising 64.3% of sequencing data. In contrast, class II elements (DNA transposons) constituted only a minor part of 1RS (5.0%). Almost the same fraction of the sequences analyzed (4.7%) showed high similarity to ribosomal RNA genes. This is consistent with the presence of the nucleolar organizing region (NOR) on 1RS.

**Figure 2:**
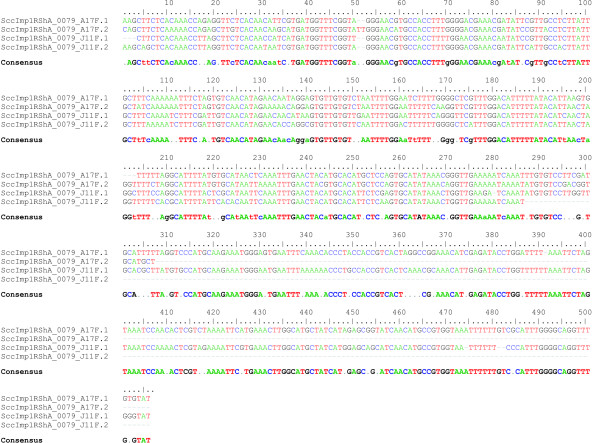
**Multiple alignment of four units of COP1 repeat**. Four units of repeat discovered in BESs SccImp1RShA_0079_A17F and SccImp1RShA_0079_J11F were aligned with ClustalW [50]. Consensus sequence was calculated from the alignment.

Searching BES with masked repeats against themselves and genome survey sequences (GSSs) from *Triticum *and *Aegilops *spp., identified 178,027 bp (8.8% of the data set) as unknown repetitive elements. However, the BESs were generally not long enough (average length 732 bp) to cover complete units of the newly identified repeats and allow the identification of new elements. Thus, most of the sequences could not be further characterized. Only one repeat, COP1 with a unit length of about 500 bp could be further characterized. Two BESs, SccImp1RShA_0079_A17F [GenBank:FI104367] and SccImp1RShA_0079_J11F [GenBank:FI104773], contained each one complete and one partial unit of this repeat with identity ranging from 85 to 95%. These sequences were used for multiple alignment (ClustalW with default settings) and consensus sequence calculation (Figure 2). Finally, a BLAST search (E-value = 1e^-10^) against the complete BES data set revealed eight complete or partial units. Assuming that our data set represents 0.5% of 1RS, one can estimate that there is more than 1,000 COP1 units in chromosome 1RS. BLAST search against NCBI nr database (E-value = 1e^-10^) revealed additional units in BAC clones from *T. turgidum *subsp.*durum *[GenBank:EF081027], *T. turgidum *subsp. *dicoccoides *[GenBank:EF067844] and *H. vulgare *[GenBank:DQ871219] with similarities ranging from 70 to 90%. In each sequence, 3 – 5 tandemly organized units were identified.

**Figure 3:**
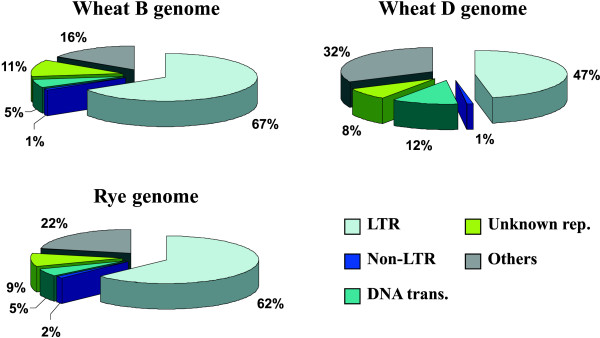
**Comparison of rye and wheat B and D genomes**. Rye genome is represented by 2 Mbp sequence of 1RS-specific BESs. Wheat B genome is represented by 10.8 Mbp sequence of random BES obtained from wheat chromosome 3B [18] and wheat D genome is represented by 2.9 Mbp sequence derived from a genomic shotgun library of the D genome progenitor *Aegilops tauschii *[36]. Note similar repeat composition of the rye and wheat B genomes.

Repeat composition of rye BESs was compared to 10.8 Mbp sequence of random BESs obtained from wheat chromosome 3B [18] and 2.9 Mbp of wheat D genome sequence derived from a genomic shotgun library of the D genome progenitor *Aegilops tauschii *[36]. The frequency of various types of repeats in rye, wheat B and D genomes revealed a close relationship of the rye genome and wheat B genome (Figure 3). To support this observation, wheat B and D genome sequences were compared to rye genome using RepeatMasker with CrossMatch algoritm. Search of 3B BESs against rye BESs masked 75.4% 3B BESs with average identity 81.3%. Search of D genome sequences against rye BESs masked only 57.6% D genome sequence with average identity 79.7%.

### Gene content analysis

Repeat-masked sequences were subjected to a homology search using BLASTN (E-value = 1e^-30^) against 960,574 plant PUTs downloaded from PlantGDB [27,28] to identify the transcribed part of the BAC end sequences. The search retrieved 93 hits. Sequences with homology to TE-related proteins were excluded from the analysis. After that, the remaining transcribed part represented 18,256 bp i.e. 0.9% of the complete sequence set. Assuming the average length of a gene in the family Poaceae to be 2 kbp, one can estimate 2,000 genes being present on chromosome arm 1RS and 36,000 genes in the whole rye genome. Forty-one sequences with alignment longer than 200 bp were searched against protein database using BLASTX. Protein with significant homology (E-value < 1e^-10^) was identified for 17 of them. Eleven of them have a putative function (Table 3).

**Table 3:** Protein homologs of predicted genes discovered in BESs from 1RS

**BES**	**Function**	**GenBank accession**	**Organism**	**E-value**
SccImp1RSbA_0223_M04F	Mla-like protein	AAR34459	*Triticum aestivum*	1e-121
SccImp1RSbA_0223_A12R	Cytochrome P450	AAK38080	*Lolium rigidum*	4e-96
SccImp1RSbA_0175_B14R	Putative nucleotide excision repair protein XP-D	NP_001054627	*Oryza sativa*	6e-96
SccImp1RShA_0079_P08R	Putative isoleucin-tRNA ligase	NP_001058191	*Oryza sativa*	8e-83
SccImp1RSbA_0223_D01R	Kinase R-like protein	AAL51071	*Triticum aestivum*	2e-74
SccImp1RSbA_0175_F17F	MLA1	AAG37354	*Hordeum vulgare*	3e-60
SccImp1RSbA_0223_P16R	Cytochrome P450	BAD37502	*Oryza sativa*	1e-56
SccImp1RSbA_0175_G24F	ACC oxidase	ABJ15735	*Triticum monococcum*	2e-63
SccImp1RSbA_0175_M02F	HCBT-like putative defense response protein	AAT38406	*Aegilops tauschii*	5e-39
SccImp1RShA_0127_E12R	Cystein proteinase	NP_001062479	*Oryza sativa*	3e-33
SccImp1RSbA_0175_G23F	Putative tRNA His guanylyltransferase	EAY98824	*Oryza sativa*	8e-29

### Development and mapping of molecular markers

Simple sequence repeats (SSRs) were identified in the data set using SciRoKo 3.3 software (see Material and Methods). In total, 216 SSRs were identified with an average length of 19.85 bp. The most abundant motifs were trinucleotides, which were found 92-times (Table 4). On average, one microsatellite was found every 9500 bp. In addition, a total of 249 sites of insertion of transposable elements were identified in the data set of 2,032,538 Mbp. Thus one may expect one transposable element insertion every 8200 bp in the rye genome. Primer pairs were designed for the 234 identified ISBPs (94.0%).

**Table 4:** Statistics of microsatellites discovered in 1RS-specific BESs

**Motif**	**Count**	**Average length (bp)**	**Count/Mbp**
Mononucleotide	19	15.89	9.35
Dinucleotide	30	29.60	14.76
Trinucleotide	92	19.99	45.26
Tetranucleotide	39	15.87	19.19
Pentanucleotide	27	16.96	13.28
Hexanucleotide	9	20.22	4.43

Total	216	19.85	106.27

**Figure 4:**
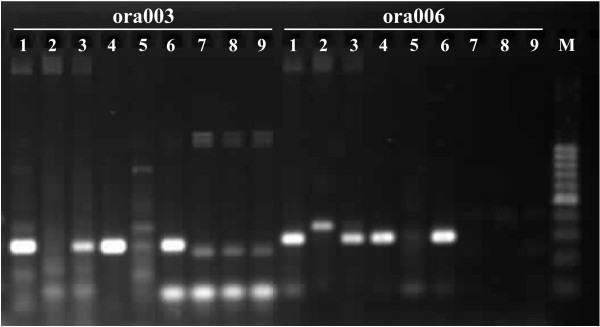
**Example of localization of ISBP markers onchromosome arm 1RS**. PCR products obtained after amplification with two ISBP primer pairs were visualized on a 2% agarose gel. Templates were DNA of rye (1), wheat (2), wheat-rye 1RS addition (3), flow-sorted chromosome 1R (4), and flow-sorted chromosomes 2R – 7R (5) were used for testing marker specificity. BAC pools of plate SccImp1RShA_0079 (6), SccImp1RShA_0127 (7), SccImp1RSbA_0175 (8), and SccImp1RSbA_0223 served to estimate frequency of ISBP sites on 1RS and as positive control. Both markers were positive in rye, the wheat-rye 1RS addition line and in flow-sorted chromosome 1R and negative in wheat and flow-sorted chromosomes 2R – 7R and thus were considered 1RS specific. M – 100 bp DNA ladder.

Sixty-four ISBP primer pairs were tested for 1RS specificity. Twelve of them (ora001 – ora012) provided an amplification product in rye and in the wheat-rye 1RS addition line but did not show any amplification with wheat DNA and thus were considered 1RS specific (Figure 4). An additional five markers (ora013 – ora017), that amplified a product from the wheat-rye 1RS addition line, were absent from rye. All 1RS-specific primers are listed in Additional file 1. Finally, ten ISBP markers were found specific for wheat. Gel electrophoresis with PCR products of the remaining 37 primer pairs resulted in bands occurring in both rye and wheat, in a smear, or had no product.

### Genomic distribution of the COP1 repeat

**Figure 5:**
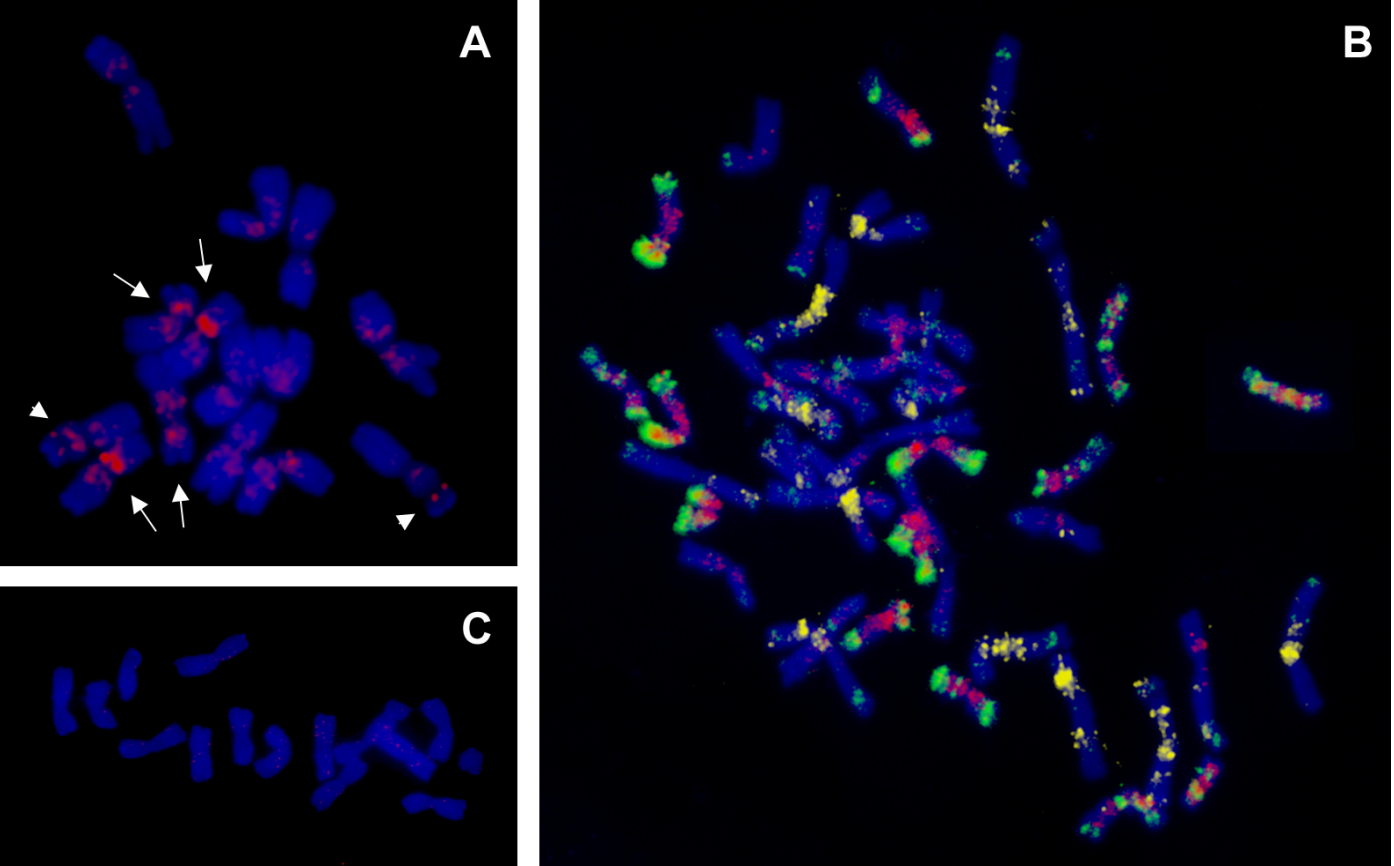
**FISH localization of COP1 repeat on mitotic metaphase chromosomes**. (A) COP1 repeat (red) is localized on three chromosome pairs of rye including the short arm of chromosome 1R (arrowheads). (B) In hexaploid wheat, COP1 shows dispersed signals on 14 chromosomes (red), belonging to the D genome, which was identified using probes for GAA microsatellites (yellow) and *Afa *family repeat (green). (C) No signal was detected after FISH with COP1 on barley chromosomes. Chromosomes were counterstained with DAPI (blue).

FISH with a probe for the COP1 repeat resulted in a signal localized on proximal end of the satellite of chromosome 1R; two other clusters localized proximally on short arms of other two chromosome pairs (Figure 5a). Thus, the COP1 repeat seems to be clustered on three pairs of rye chromosomes. In spite of using blocking DNA prepared from sheared genomic rye DNA, cross-hybridization was detected on all rye chromosomes. FISH in hexaploid wheat revealed dispersed signals over 14 chromosomes (Figure 5b), indicating that COP1 is dispersed in one of the three wheat homoeologous genomes. Reprobing with probes for GAA microsatellites and *Afa *repeat proved that the repeat is localized on the D genome chromosomes. No signal was detected after FISH with the COP1 repeat on barley metaphase chromosomes (Figure 5c).

## Discussion

We generated and analyzed 2 Mbp of BAC end sequences from the short arm of rye chromosome 1 (1RS) accounting for 0.5% DNA of the arm. This study provides the largest amount of genomic sequence data for rye and allows the first systematic analysis of the DNA sequence composition of the rye genome. Because the BAC clones selected for end sequencing were chosen randomly and originated from BAC libraries constructed with two different restriction enzymes, the BESs produced here are expected to be randomly distributed along the whole chromosome arm. Assuming that there is no or little difference in sequence composition among different rye chromosomes, one can consider this sequence as representative of the whole rye genome. The GC content of 1RS (45.9%) is comparable to 44.5% and 44% GC content in wheat (estimated from 37,609 BES downloaded from GenBank [31]) and rice genomes [9], respectively.

The observed content of repetitive sequences (84.2%) is lower than expected and is similar to that found in the wheat B genome (85.9%) by Paux et al. [18]. As indicated above, rye has a significantly higher 1Cx value than bread wheat. Thus, assuming an equal number of genes, the rye genome should contain more repeats than the wheat genome. In fact, using Cot analysis, Flavell et al. [8] estimated the content of repetitive elements in rye genome to be of about 92%. Our low estimate could be due to insufficient representation of rye repeats in databases that were used in our analyses. For example, the TREPtotal database, which showed most of the significant matches, comprises only 39 entries of rye repeats. This limited information, compared to wheat and barley with 663 and 554 elements, respectively (in the same database), could result is short sequence alignments and hence underestimation of the amount of repeated DNA in rye BES. As expected, Class I elements (retrotransposons) were the most abundant repetitive fraction in the rye genome similar to what was found for the wheat and maize genomes [18,37].

A comparison of the frequency of various types of repeats in genomes of rye and wheat B and D genomes suggested a greater similarity between the rye and wheat B genome than between the rye and wheat D genome. This close relationship of rye and wheat B genome was also supported at the sequence level. It is interesting to note that rye and the putative B genome progenitor *Aegilops speltoides *have the same mating system, both being outcrossers. Moreover, the B genome is largest of the three homeoelogous wheat genomes and similar in size to the rye genome. On the other hand, the similarity in repeat composition between both genomes may simply reflect similar trends in the mode of their expansion via the LTR-retrotransposon activation.

Until now, the lack of sequence data did not permit estimation of the number of genes in rye. By analyzing 2 Mbp sequence from chromosome arm 1RS, we estimate 2,000 genes being present on 1RS and thus 36,000 genes in the rye genome. This first estimate for rye is consistent with the predicted gene numbers in other plants. Most recent estimate for gene number in *A. thaliana *genome is 33,000 (TAIR8 release) [38], the current TIGR rice genome annotation (Release 5) [39] estimates 41,000 genes in rice genome. Both numbers are close to our prediction for rye.

In addition to the analysis of the rye genome composition, we used BAC end sequences for marker development. There is still a low density of markers available for rye genome and additional markers are urgently needed. Development of a genetic linkage map of rye with 183 markers was reported by Korzun et al. [40]. Bednarek et al. [41] presented a genetic map of rye containing 480 markers including 200 RFLPs, 179 AFLPs, 88 RAPDs and 13 proteins. Khlestkina et al. [42] mapped 99 SSRs derived from EST sequences (SSR-ESTs), nine of which mapped to chromosome 1R. Several attempts were made to transfer SSR and/or EST-SSR markers from wheat and barley into rye [43-45]. Recently Varshney et al. [46] succeeded in transferring and mapping 12 barley SNP markers in rye. Thus, to date the density of markers is quite low and does not allow efficient map based cloning or MAS in rye.

This work presents a method for targeted development of molecular markers from specific parts of the rye genome using the 1RS chromosome arm as a case study. Until now, there are only a few markers available for this critical part of the rye genome. This hampers marker assisted breeding not only in rye, but also in wheat where the markers from 1RS would permit selection of lines with introgressions of desired parts of 1RS without the harmful genes. We have developed twelve new ISBPs markers for 1RS and designed almost 200 additional primer pairs for potential ISBP markers that remain to be tested. If needed, generation of additional BAC end sequences from 1RS is possible. In addition to the development of new ISBP markers, BES containing microsatellites were used to develop new 1RS-specific SSR markers. Out of the 63 tested microsatellites that were tested, 21 were specific for 1RS [47]. Thus, in total 33 1RS-specific markers were obtained using our strategy. On the other hand, Badnarek et al. [41] isolated 198 AFLP and RAPD markers from genomic DNA and only 29 markers were specific for 1RS. This clearly demonstrates the efficiency of the targeted approach.

One of the potential problems in targeted marker development from the 1RS-specific BAC library is the contamination with clones from wheat DNA. This could explain the specificity of amplification for several ISBP markers with wheat DNA. As the 1RS arm was flow-sorted from a wheat-rye ditelosomic addition line, contamination by fragments of wheat chromosomes cannot be avoided. However, the contamination of the 1RS BAC library was estimated to be only 14% [15] and thus should not seriously compromise the efficiency of marker development. Further improvement could be achieved by selecting BAC clones from contigs after fingerprinting the library, as the contigs originate from the chromosome of interest while the infrequent contaminating BACs remain as singletons.

The discovery of five ISBP markers, which were specific only for 1RS maintained in the wheat-rye ditelosomic addition line and were not found in diploid rye, was unexpected. Although the same cultivars (i.e. Chinese Spring and Imperial) were used for the development of the 1RS wheat-rye addition line as well as for marker testing, these insertion sites were absent from rye and wheat. This suggests that some mobile elements were activated only after the addition of 1RS telocentric to wheat, most probably as a consequence of interspecific hybridization. Among the five activated elements three are retrotransposons (Copia-like, LINE and SINE) and two are DNA transposons (MITE and Mutator). Liu and Wendel [48] and Shan et al. [49] observed similar activation of both classes of transposable elements after a hybridization of cultivated (*Oryza sativa*) and wild (*Zizania latifolia*) rice.

## Conclusion

This work provides the first insights into the composition of the rye genome and its chromosome arm 1RS, in particular. We demonstrate that the use of chromosome arm-specific BAC libraries facilitates the analysis of complex plant genomes by targeting particular genomic regions as well as by developing molecular markers for these regions. New molecular markers from 1RS should help in saturating the genetic map of 1RS, and aid marker assisted breeding and gene cloning.

## Abbreviations 

1RS: short arm of rye chromosome 1; BAC: bacterial artifical chromosome; BES: BAC end sequence; ISBP: Insertion Site Based Polymorphism; PUT: PlantGDB-assembled Unique Transcripts

## Authors' contributions

JB, EP and RK participated in DNA sequence analysis. JB and MH mapped newly isolated ISBP markers. DK performed the FISH experiments. PS sorted chromosomes using flow cytometry. JŠ and HŠ made an intellectual contribution to the concept of the experiment. CT sequenced BAC ends. JB drafted the manuscript. TL and CF revised manuscript critically for important intellectual content, JD conceived and supervised the project and prepared the final version of the manuscript. All authors read and approved the final manuscript.

## Supplementary Material

Additional file 1 List of 1RS-specific ISBP markers Click here for file
