# Supplementary material for: A first survey of the rye (Secale cereale) genome composition through BAC end sequencing of the short arm of chromosome 1R
Source: BMC Plant Biol. 2008 Sep 19;8:95. doi: 10.1186/1471-2229-8-95 (PMC2565679; doi:10.1186/1471-2229-8-95)
Supplement: Additional file 1 — List of 1RS-specific ISBP markers [file 1471-2229-8-95-S1.pdf]

List of 1RS-specific ISBP markers

Marker*	BES	Left primer	Right primer	Annealing temp. (°C)
ora001	SccImp1RShA_0079_A08R [GenBank:FI104512]	TCCGCTCTTCGTGCTCCC	GCATCCCCTCTTTCGTCTTCATC	62
ora002	SccImp1RShA_0079_A21R [GenBank:FI104372]	TCCACTTTCCTCCCACTCACTTC	ACCGACACGTTCGTTGCTG	62
ora003	SccImp1RShA_0079_H12F [GenBank:FI104934]	GGAAATGCCAACGCGGATCAC	GCCTGCTCTATGCCGACAC	62
ora004	SccImp1RShA_0079_I14F [GenBank:FI104609]	TTGAATGGCTCCAAAGGAAC	TTACGGACGCATTTGTCTGA	62
ora005	SccImp1RShA_0079_J05R [GenBank:FI104768]	TTGGTCCTCCTTGAAATTGG	TTAGCTCTTTGGGCTTTGGA	62
ora006	SccImp1RShA_0079_J12R [GenBank:FI104958]	CATGGGATCGCTCTCGAAGAAC	TCGAAGCCCAAGCAATATCACAC	62
ora007	SccImp1RShA_0079_L17R [GenBank:FI104804]	CGCATCGACAAGCTCGTAACC	TGTGCCCCAATCACGTATTCATC	62
ora008	SccImp1RShA_0127_K13R [GenBank:FI105165]	CGGCAACACCTTTCAATATGCAC	ATGGAACACTTCTACGACAGCAC	62
ora009	SccImp1RSbA_0175_D16R [GenBank:FI106021]	GGCGAAGCCGATGAGACTCTG	CCCCCGCCGAACCGAAAG	62
ora010	SccImp1RSbA_0175_P21R [GenBank:FI106001]	GATAGTAGCAACAGTGTGGTGGG	CAGAGAAGCAAGGAAAGGTGGTG	62
ora011	SccImp1RSbA_0223_E09F [GenBank:FI106499]	CGCCGTAGTCTGATCGAGCC	GTCTCCCGTGCGTATTTTTCTCC	64
ora012	SccImp1RSbA_0223_E11R [GenBank:FI106502]	GCACTGTGGTGCACTGAGTT	GAACTCCACCACCTCCTTGA	64
ora013**	SccImp1RShA_0079_B18F [GenBank:FI104871]	TGTTATATGAGCGCGAACCA	GCAGAAGTTGGGCGTGTACT	62
ora014**	SccImp1RShA_0079_F20R [GenBank:FI104919]	AACTCCGAATCGTTGGGATA	CCGTCGTCCCAAAATAGTGT	62
ora015**	SccImp1RShA_0127_C24R [GenBank:FI105241]	GCCGTCGTCTTCCTGAATAG	ACCAAGATGAGCACCAAACC	62
ora016**	SccImp1RSbA_0175_E09R [GenBank:FI105757]	TCCGTCCCCGTCTCCGTC	ATAAGCCGTGCAAGTCGCC	62
ora017**	SccImp1RSbA_0223_K19R [GenBank:FI106575]	TGACAAATTGGTTCGCAAGGGG	GCAGCCGTCCACAGACATATAG	62

*ora = Olomouc rye ISBP marker
**ora013 – ora017 are specific for 1RS in wheat-rye addition line, and are absent from 1RS in rye (for details see discussion).
